# Emotions and interactive tangible tools for math achievement in primary schools

**DOI:** 10.3389/fpsyg.2024.1440981

**Published:** 2024-10-29

**Authors:** Filippo Saccardo, Gisella Decarli, Valentina Irene Missagia, Margherita Andrao, Federica Gini, Massimo Zancanaro, Laura Franchin

**Affiliations:** ^1^Department of Psychology and Cognitive Science, University of Trento, Trento, Italy; ^2^Fondazione Bruno Kessler, Trento, Italy

**Keywords:** math learning, emotions, tangible tools, children, primary education

## Abstract

**Introduction:**

Acquiring mathematical concepts is crucial for students’ academic achievements, future prospects and overall well-being. This study explores the role of emotions in a symbolic number comparison task and the impact of the use of a tangible tool.

**Methods:**

Fifty-nine healthy children aged 6 to 7 years participated in a between-subject study with two conditions for the modality, digital tools vs the use of pen and paper, and two conditions for emotions, positive vs neutral.

**Results:**

The study provided evidence that positive emotions can improve task efficiency for pen and paper modality, and the use of the digital tool improves task efficiency with both positive and negative emotions.

**Discussion:**

These findings suggest that addressing emotional factors before engaging in a symbolic task can enhance learning and that interactive technology may give a more significant benefit to students with less positive attitudes toward the task. Incorporating effective teaching methodologies that utilize tangible devices within a positive emotional context can foster engagement and achievement in mathematics, optimizing students’ learning experiences.

## Introduction

The acquisition of mathematical skills is a cornerstone of education and has a profound impact on students’ academic progress and future prospects (OECD, 2016). However, the path to mastering mathematical concepts and skills poses significant cognitive challenges. The process relies on multiple cognitive mechanisms and unfolds across critical developmental milestones. Some authors have suggested that the acquisition of math is connected to numerical processing, which encompasses both symbolic and non-symbolic representations (Dehaene, 2011).

Representing and manipulating numerical magnitudes is critical for understanding early mathematical concepts (Decarli et al., 2023b; Starr et al., 2013). Remarkably, this ability manifests from the earliest stages of life (Izard et al., 2009) and is intertwined with multiple cognitive domains (Decarli et al., 2022a; Decarli et al., 2022b). Moreover, impairments in the discrimination of non-symbolic numerosities characterize developmental dyscalculia, a specific learning disability within the mathematical domain (Piazza et al., 2010). Equally important is the domain of symbolic knowledge: the mastery of symbols occupies a crucial position in mathematical learning and shows significant deficits in children with dyscalculia (Noël and Rousselle, 2011; Rousselle and Noël, 2007). A wealth of literature confirms the central role of symbolic/non-symbolic processing in both typical and atypical mathematical acquisition (e.g., Decarli et al., 2023a; Honoré and Noël, 2016; see Schneider et al., 2017 for a meta-analysis).

Moreover, the process of acquiring mathematical knowledge goes beyond mere cognitive functions; emotional facets also exert a profound influence (Doctoroff et al., 2016; Sainio et al., 2019). Understanding the intricate interplay between mathematical learning and emotions is essential for promoting positive learning experiences, fostering motivation, and optimizing educational outcomes in mathematics (Schukajlow et al., 2017).

Many students experience a spectrum of emotions as they engage with mathematical concepts and tasks. Negative emotions such as anxiety, frustration, and feelings of helplessness are prevalent during these learning experiences (Pekrun, 2014). These emotions have a significant impact on students’ motivation, engagement, and overall academic performance in mathematics (Wang et al., 2020). For example, many studies have highlighted the negative effects of math anxiety on mathematics learning (see, e.g., Ashcraft and Krause, 2007; Ramirez et al., 2013). Math anxiety, characterized by apprehension or fear of mathematics, triggers heightened physiological arousal and cognitive interference (Hembree, 1990). Its presence reduces students’ working memory capacity, hinders problem-solving abilities, and ultimately undermines mathematical achievement (Barroso et al., 2021; Ramirez et al., 2016).

Emotions also influence various aspects of learning motivation. Specifically, students’ positive emotions support their beliefs in the incremental theory of intelligence and increase the confidence in their intellectual abilities (Acosta-Gonzaga, 2023; Pesu et al., 2016). Furthermore, positive emotions significantly impact students’ perceptions of their academic capabilities and their pursuit of mastery-approach goals, aligning with the predictive nature of achieving academic objectives (Mega et al., 2014).

Emotions have a significant impact on math learning and have a complex effect on students’ cognitive processes, attention, and problem-solving abilities (Pekrun et al., 2011). Students’ emotional states are intertwined with their self-regulated learning and motivation, with profound implications for academic performance (Mega et al., 2014; Pekrun et al., 2011). Effective emotion regulation strategies, such as cognitive reappraisal and self-regulation, hold promise for alleviating negative emotional experiences associated with mathematical learning (Gross, 1998). Educating students about emotion regulation and adaptive responses can increase engagement, reduce anxiety, and cultivate a positive learning environment.

The role of teachers’ support and classroom climate proves critical in shaping students’ emotional experiences during mathematical learning (Schonert-Reichl, 2017). Positive teacher-student relationships, clear explanations, and collaborative learning opportunities contribute to a nurturing and motivating atmosphere, that promotes emotional well-being and effective mathematical learning (Rimm-Kaufman et al., 2009). In addition, integrating socio-emotional learning programs into mathematics education shows potential for improving students’ emotional literacy and mathematics achievement (Durlak et al., 2011).

Understanding the complex interplay between mathematical learning and emotions is a critical part of designing effective educational practices that support students’ emotional well-being and optimize their mathematical learning experiences. By acknowledging the emotional landscape alongside the cognitive developmental processes involved, educators can foster positive emotions, increase engagement, and improve achievement in mathematics. Taken together, these findings suggest that the use of concrete learning approaches to mathematical skill acquisition (Swanson and Williams, 2014; Zulfakri et al., 2019), coupled with positive emotional support, can strengthen children’s emotional states and subsequently their learning.

Tangible, interactive tools are gaining attention in education for their potential to support multiple facets of the learning process (González-González et al., 2019). Several studies have investigated the benefits of employing tangible objects to teach mathematical concepts (Nagaraju and Jain, 2015; Ueno, 2017; Zito et al., 2021). As observed by Inhelder and Piaget (1964), physical interaction with the environment allows children to grasp abstract concepts more concretely, fostering learning and cognitive development. Moreover, tangible technologies have the potential to enhance playful learning, engagement, and reflection (González-González et al., 2019). Recently, educators and researchers have shown interest in adopting tangible smart devices to support the learning-teaching process (Al-Emran et al., 2020; Moreira F. T. et al., 2017). Smart devices provide opportunities for children to build knowledge in engaging and interactive ways (Forman and Pufall, 1988). Moreover, they may aid teachers by providing real-time information on students’ learning processes and performance (Al-Emran et al., 2020; Moreira F. et al., 2017; Moreira F. T. et al., 2017).

The aim of the present study was twofold. First, we aimed to investigate the relationship between induced emotional states and mathematical learning by examining the impact of emotions prior to task engagement. Second, we sought to evaluate the effectiveness of using interactive tangible technology compared to traditional paper-and-pencil methods. The interactive tangible tool used was SMARTER (Andrao et al., 2022), a box equipped with a microcontroller and five RFID sensors. This system is programmable in the mathematical domain to perform arithmetic tasks using symbolic tiles. Our hypotheses were as follows:

*Hp1.* We hypothesize that positive emotional elicitation will lead to better mathematical performance compared to neutral emotional states;

*Hp2.* We hypothesize that the use of the interactive tangible tool (SMARTER) will result in greater improvements in mathematical achievement than the traditional paper-and-pencil method;

*Hp3.* We expect that the combination of positive emotion induction and the SMARTER tool will produce the most significant gains in math performance, suggesting a synergistic effect between emotional states and interactive learning tools.

Previous research in education, psychology, and human-computer interaction has often separated different aspects or used tangible devices primarily to enhance emotional expression in social and affective interactions (e.g., Gooch et al., 2022; Vansan Gonçalves et al., 2021; Wallbaum et al., 2017). The novelty of our study lies in the intentional manipulation of emotions in conjunction with novel learning technologies, embedding them at the core of the learning environment. This approach challenges the traditional paradigm of using tangible devices solely to enhance emotional expression, while ignoring the emotional state of the participant.

## Methods

### Participants

Fifty-nine healthy children aged 6 to 7 years (*M*age = 6.6 years; *SD* = 0.3) participated in the study. All participants were recruited from the first year of a primary school located in the North of Italy. All children who provided informed consent from their parents were included in the sample. The exclusion criteria were a diagnosis of learning disorders or other developmental disabilities. The study was approved by the Ethical Committee of the University of Trento and all procedures were conducted in accordance with the ethical standards laid down by the Declaration of Helsinki. Specifically, in our ethical approval document, we addressed the potential for distress caused by the experimental conditions. First, we ensured that the procedure did not expose participants to any psychological or physical risks. Second, to mitigate any potential unpleasant or stressful effects from viewing emotional videos, we planned the presentation of a funny video after the post-training phase to create a positive atmosphere at the end of the experiment. Additionally, experimenters were instructed to comfort any child who did not respond positively to the video through engaging and enjoyable activities, such as games.

To be included in the study, participants were required to obtain scores within normal limits on the Coding and Digit Span subtests of the WISC-IV, administered prior to the testing phase. Additionally, participants needed to understand the Italian language to be able to follow the instructions given by the experimenters. Only one child was excluded for not meeting this language requirement. Children were randomly assigned to the *SMARTER* group (*n* = 31; 13 females; *M*age = 6.5 years; *SD* = 0.29) or to the paper-and-pencil group (*n* = 28; 14 females; *M*age = 6.6 years; *SD* = 0.32). The two groups did not differ in terms of age, *t*(57) = −1.56, *p* = 0.12, or gender, χ2(1) = 0.13, *p* = 0.72. Children in the two groups had a similar IQ, as estimated from the combination of the Digit Span and the Coding subtests of the WISC-IV (Wechsler, 2003; *t*(56) = −0.41, *p* = 0.68). Moreover, participants were assigned to one of the two emotional conditions (positive: *n* = 34; 15 females; neutral: *n* = 25; 12 females) and they did not differ in terms of age, *t*(57) = 0.91, *p* = 0.37, IQ, *t*(56) = 1.09, *p* = 0.28, nor gender, χ2(1) = 0.42, *p* = 0.51.

### Procedure

**Figure 1:**
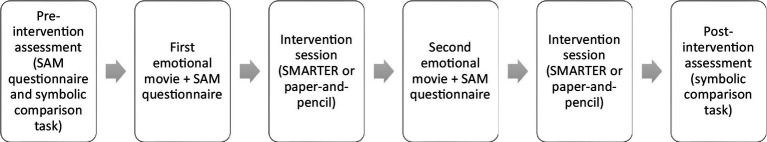
Schematic representation of the procedure. The procedure was identical for all the groups. The core part of the procedure (emotional movie- SAM questionnaire- intervention session) was repeated twice.

The experimental session took place in the quiet computer-room of the school and participants completed the *SMARTER*/paper-and-pencil activity divided into small groups (max 3 children per group) under the experimenters’ supervision. A schematic representation of the procedure is presented in Figure 1. During the pre-intervention phase, all participants completed the Self Assessment Manikin questionnaire (SAM; Bradley and Lang, 1994; Lang et al., 2005) to assess their emotional state and they were asked to complete the symbolic number comparison. Afterwards, children were shown two movie clips (see Supplementary Table S1 for the complete list of movies), according to the condition they were assigned (positive or neutral). The movie clips were projected on an interactive whiteboard, and children were asked to rate how each clip made them feel by using the SAM.

Thereafter, the intervention sessions began. Participants in the *SMARTER* group completed the first activity session by using the tangible tool, whereas children assigned to the paper-and-pencil group completed an equivalent activity (see the Intervention sections described below). This first session was followed by a second activity session. The decision to include a second intervention session was primarily driven by the aim of providing children with more learning opportunities. Moreover, this additional session was implemented to increase children’s involvement. Finally, they were asked to complete the post-intervention evaluation. At the end of the testing phase, an additional positive video was shown in order to conclude the experiment in a positive emotional state.

#### Elicitation and evaluation of emotional states

Four standardized emotional videos of 3 min each were selected from previous studies, two of these to elicit positive emotions, and two for neutral emotions (de Freitas Brandão et al., 2016; Essex et al., 2003; von Leupoldt et al., 2007; Talge et al., 2008; see Supplementary Table S1). The Italian version of the clips was used and the order in which they were presented to the groups was randomized.

In order to evaluate the children’ emotional state, the SAM questionnaire (Bradley and Lang, 1994; Lang et al., 2005) was administered. SAM is a paper and pencil questionnaire that allows for subjective evaluation by participants of their perceived emotional experience based on the dimension of pleasure. This instrument consists of three 9-point graphical scales. The different scale points are represented by stylized little men, presented as “SAM,” whose expressions indicate one different type of feeling, happy/unhappy. SAM thus varies from a smiling and happy figure to a frowning and unhappy figure when representing the valence dimension. There is also the opportunity to choose intermediate positions between one little man and the other. Participants completed the first SAM as a baseline mood measure and after the 2 emotional videos, for a total of 3 evaluations.

#### Pre- and post-intervention task

Participants were presented with 2-digit numbers ranging from 21 to 40 and were asked to insert the correct symbol (either “>“or “<“) between them. Ten number pairings (21–40, 37–39, 28–31, 30–40, 25–34, 23–21, 35–26, 38–34, 33–20, 27–25) were selected to include both easier and more difficult number couples. Indeed, participants were taught how to compare quantities using numbers from 0 to 9 as part of the curriculum unit before the experiment. Performance was measured in terms of accuracy and reaction times (RTs in seconds).

#### Intervention for the SMARTER group

*SMARTER* is designed to foster teaching-learning environments by providing an interactive and engaging setting suited for learning math in primary school children. *SMARTER* consists of a plywood box with a surface of 26 × 11 cm where five rectangle slots (4 × 5 cm each) are carved to allow the placement of plywood tiles of 3 × 4 cm with RFID tags embedded. The tiles represent operators (+, −, ×, ÷), symbols (=, <, >), and digits (from 0 to 9). This tool contains a small computer board that controls 5 RFID readers placed under each slot, an RGB LED for visual feedback, and a speaker for audio feedback (see Supplementary Figures S1–S3).

*SMARTER* can be programmed to support different math exercises and provide specific visual and audio feedback when the exercise is correctly, or incorrectly completed by the child, or not completed. The *SMARTER* device is designed to be autonomously programmed by teachers, although this aspect is beyond the scope of this paper (for more details on the tool see Andrao et al., 2022, 2023): for this study, the SMARTER device has been pre-programmed to implement the same tasks used in the paper-and-pencil condition.

A *SMARTER* device was used to provide a “green” led light feedback when digit tiles were added on the surface and a “blue” led light when they placed the symbol whether they correctly or incorrectly completed the exercise. For this exercise, the feedback about the correctness of the exercise was provided by the operator to prevent any potential, repeated negative feedback from the tangible device from negatively altering the child’s emotional state.

#### Intervention for the paper-and pencil group

Children were shown a booklet with two-digit numbers comparison tasks to do with the paper and pencil. During the intervention phase, they were taught several calculation strategies to improve their performance and correct mistakes. For instance, to determine the largest number, children were encouraged to focus on the “tens” digit first and, only if they were identical, consider the “units” digit. Children received personalized feedback from the experimenters about their performance in both conditions, SMARTER and paper-and-pencil.

#### Experimental design

In the present study, we implemented a randomized controlled trial design. Indeed, participants were randomly divided into 4 groups: *SMARTER* and positive emotion (*n* = 19), *SMARTER* and neutral emotion (*n* = 12), paper-and-pencil and positive emotion (*n* = 15), paper-and-pencil and neutral emotion (*n* = 13). This design allows us to investigate the effects of the type of intervention (SMARTER vs. paper-and-pencil) and the emotional condition (positive vs. neutral) on mathematical performance. We performed *t*-tests to compare performances in each experimental condition. Additionally, we conducted Wilcoxon tests because the data were not normally distributed in most comparisons, and the sample size was relatively small.

### Data analysis

For each child, we calculated an inverse efficiency score (RTs/Accuracy; see Decarli et al., 2023a; Lyons et al., 2014; Sasanguie et al., 2017). The combination of RTs and accuracy in one unique index is particularly suitable to assess the performance, obtaining a more comprehensive understanding of overall performance in pre- and post-intervention.

## Results

### Effect of training on performance

**Figure 2:**
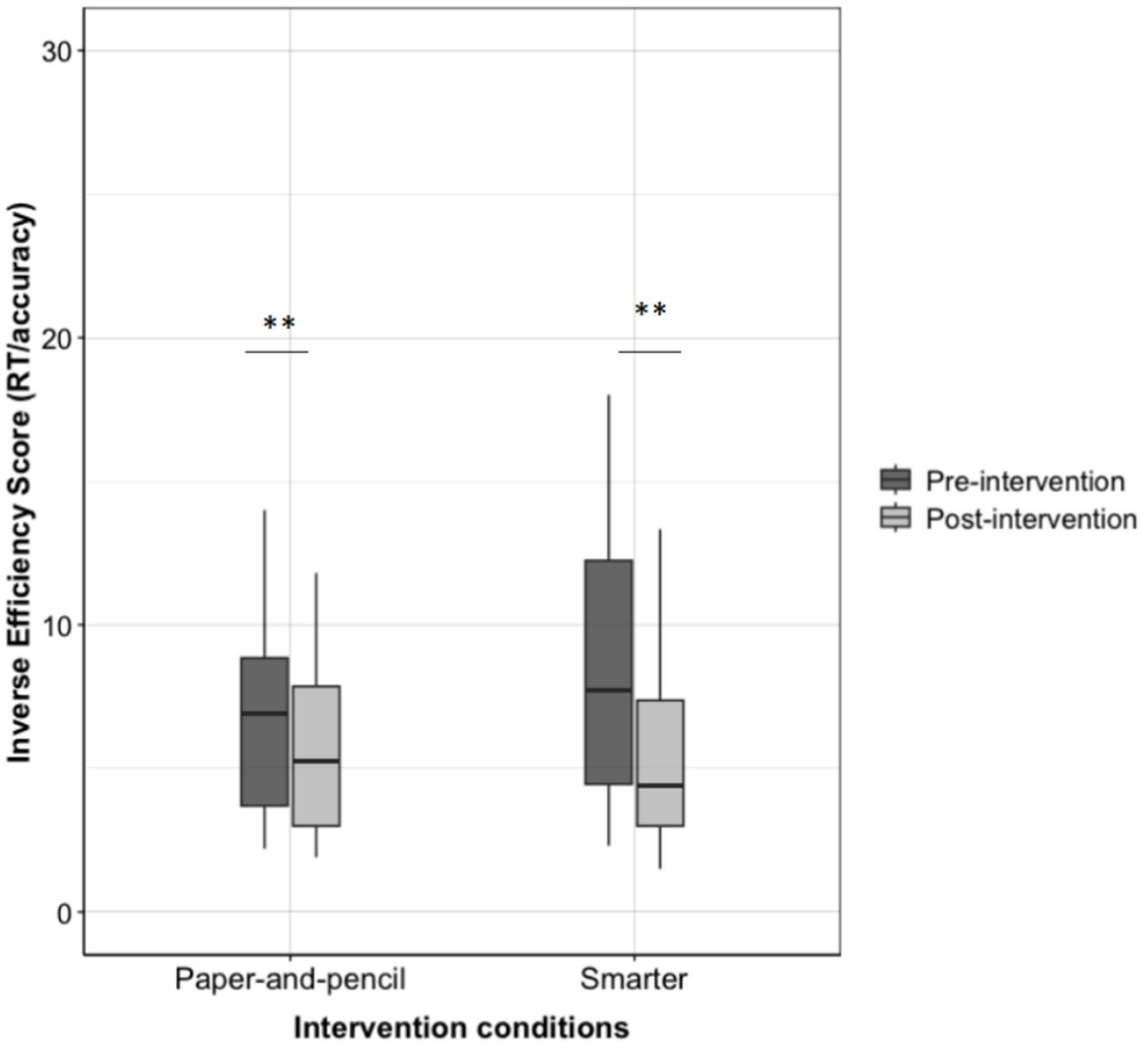
Inverse efficiency score (RT/accuracy) for pre- and post-interventions (SMARTER vs. paper-and-pencil group).

Both the *SMARTER* group (pre-intervention: *M* = 9.25, *SD* = 6.56; post-intervention: *M* = 6.48, *SD* = 5.51) and the paper-and-pencil group (pre-intervention: *M* = 7.00, *SD* = 4.02; post-intervention: *M* = 5.6, *SD* = 2.98) showed significant improvements in the performances, *t*(30) = 2.65, *p* = 0.01; *V* = 441, *p* < 0.001 for the *SMARTER* group, *t*(27) = 3.03, *p* = 0.005; *V* = 336, *p* = 0.002 for the paper-and-pencil group (see Figure 2).

In the SMARTER condition, involving two intervention sessions, we conducted an analysis to assess the improvements between the first and second session. Notably, a significant difference emerged between the two sessions (*M* = 2.72, *SD* = 1.59 for session 1; *M* = 1.8, *SD* = 1.01 for session 2; *t*(25) = 5.11, *p* < 0.001; *V* = 333.5, *p* < 0.001), highlighting meaningful improvements over the course of the intervention.

### Effect of emotion on performance

We analyzed the effect of emotions on the children’s performance. Before the projection of the movies, children presented the same level of happiness (positive emotion_baseline_: *M* = 1.88, *SD* = 1.85; neutral emotion_baseline_: *M* = 1.64, *SD* = 1.25; *t*(57) = 0.56, *p* = 0.57; *W* = 431, *p* = 0.91). However, after the movies’ projections, children in the positive emotion group maintained this emotional state (*M* = 2.16, *SD* = 1.73; *t*(33) = −0.74, *p* = 0.46; *V* = 41.5, *p* = 0.30), while the neutral group reported significant lower scores of happiness (*M* = 2.74, *SD* = 1.77; *t*(24) = −2.86, *p* = 0.009; *V* = 19.5, *p* = 0.01). We can interpret these results in light of the fact that children start from a baseline situation in which they reported a high level of happiness. The administration of positive videos did not have significant effects, and did not make the children even happier, while the viewing of neutral videos significantly shifted the mean toward a state of decreased happiness.

**Figure 3:**
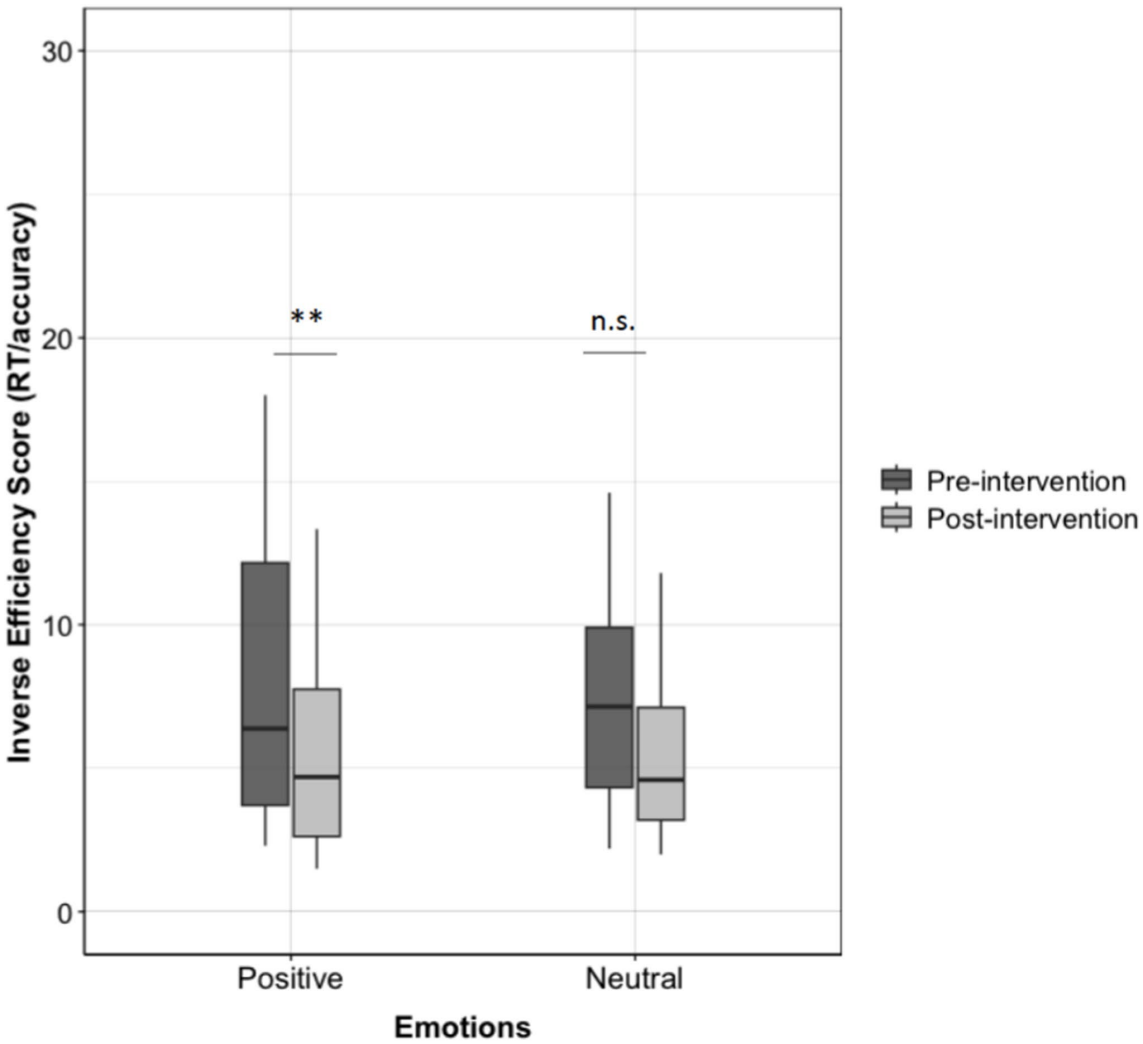
Inverse efficiency score (RT/accuracy) for pre- and post-interventions for different emotions (positive vs. neutral).

Furthermore, we analyzed the pre- and post-intervention in the two emotional conditions. The results showed that only positive emotions led to better performances in the post-intervention (pre-intervention: *M* = 8.72, *SD* = 6.7; post-intervention: *M* = 6.04, *SD* = 4.45; *t*(33) = 3.28, *p* = 0.002; *V* = 539, *p* < 0.001) and partially, not the neutral ones (pre-intervention: *M* = 7.46, *SD* = 3.51; post-intervention: *M* = 6.09, *SD* = 4.6; *t*(24) = 1.62, *p* = 0.12; *V* = 270, *p* = 0.003; see Figure 3).

To explore our data more in-depth, we analyzed the pre- and post-intervention performances across different emotions and training conditions. Since the sample size was really small and the data do not follow a normal distribution, we performed only non-parametric Wilcoxon tests. We found significant differences between pre- and post-intervention scores in the *SMARTER* condition for both positive emotions (pre-intervention: *M* = 9.88, *SD* = 7.99; post-intervention: *M* = 6.29, *SD* = 5.46; *V* = 175, *p* < 0.001) and neutral emotions (pre-intervention: *M* = 8.25, *SD* = 3.32; post-intervention: *M* = 6.79, *SD* = 5.83; *V* = 66, *p* = 0.03). However, in the paper-and-pencil intervention, we found significant differences only for positive emotions (pre-intervention: *M* = 7.25, *SD* = 4.42; post-intervention: *M* = 5.73, *SD* = 2.88; *V* = 106, *p* = 0.007) but not for neutral emotions (pre-intervention: *M* = 6.72, *SD* = 3.66; post-intervention: *M* = 5.44, *SD* = 3.21; *V* = 71, *p* = 0.08).

## Conclusion

The existing literature already illustrates the beneficial effects of positive emotions on students, leading to improved academic performance, increased motivation to learn, and increased engagement (Mega et al., 2014; Pekrun, 2014). In our study, we explored the role of emotions in the learning process by exposing one group of children to positive movie clips and another to neutral clips. The results confirmed the positive influence of manipulating emotions on learning (in line with our first hypothesis). Consequently, our findings highlight the importance of fostering a positive school environment, especially in academic domains such as math, which are known to induce high levels of anxiety (e.g., Ashcraft and Krause, 2007; Ramirez et al., 2013, 2016).

Our second goal was to assess the effectiveness of using interactive, tangible technologies to enhance learning, with a particular focus on symbolic math skills. The children were divided into two subgroups: one using the traditional paper-and-pencil method for symbolic number comparison, and the other using SMARTER for the same task. This division allowed us to compare the two learning methods (SMARTER vs. paper-and-pencil). Contrary to our second hypothesis, the analyses revealed significant pre and post differences in both conditions. In the SMARTER condition, children showed increased interest in the new interactive tool. SMARTER showed excellent usability, allowing children to use its potential without specific training. However, sustained use of the tool may be necessary to observe more substantial improvements compared to the paper-and-pencil method. In addition, the initial intervention phase showed satisfactory levels of accuracy, suggesting that the task may have been relatively easy for the children.

To delve deeper into our data, we conducted a comprehensive analysis of pre- and post-intervention performance, controlling for emotion and training condition. Separate analyses for positive and neutral emotions showed significant improvements for both emotional states in the SMARTER condition. In contrast, the paper-and-pencil intervention showed significant improvements only for positive emotions. These results suggest that the SMARTER intervention may be more effective in improving mathematical performance across a broader range of emotions, including both positive and neutral states. Additionally, it is important to consider that the mere introduction of the SMARTER tool may have influenced other psychological components, such as curiosity, motivation, and engagement. These factors could have played a role in mediating the improved performance observed in the SMARTER intervention, regardless of the emotional state (positive/neutral). While our study focused on emotional states using the SAM questionnaire, future research should explore how technological tools like SMARTER can impact other components beyond positive or neutral states. Understanding these broader emotional influences could provide deeper insights into how interactive tools can enhance learning outcomes across diverse emotional contexts.

In future studies, this tangible tool could be used for calculations involving basic operations and could be expanded by pairing it with another SMARTER device to handle larger numbers and more challenging tasks. The lack of challenge in the tasks for the participants in this study serves as a limitation to be considered in future research, which could be extended to other academic areas, such as linguistic tasks. The tool’s tiles could be programmed to represent syllables or words, facilitating word or sentence formation tasks. Its scalability allows it to be used in different learning domains. The development of programming interfaces and gamification will allow teachers to create specific tasks independently, providing students with a more engaging learning experience. In addition, the methodology used in this study can be extended to the use of other physical devices and analyzed over longer periods of time to test the impact of new teaching methods on learning. One limitation of the present study is that our sample size could have been larger and more diverse to enhance the generalizability of the results. Future research can expand our findings by including a broader range of participants and exploring the effectiveness of these tools in different contexts. Additionally, while our study focused primarily on the direct impact of the SMARTER tool on students’ performance and emotional responses, we recognize that the teacher’s role in facilitating the use of technology and managing classroom emotions is a critical element that was not addressed in our research. Future studies should explore the influence of teachers’ perceptions, training, and interactions with these technologies to better understand their impact on student learning and emotional experiences.

By recognizing the emotional aspect of mathematics learning and understanding cognitive developmental processes, educators can cultivate positive emotions, engagement, and achievement in mathematics. Concrete approaches to learning mathematical skills, coupled with emotional support, have been shown to improve children’s emotional states and, consequently, their learning. Our study highlights the role of positive emotions in learning from the earliest stages of schooling. It also demonstrates how the integration of tangible tools into teaching methods can enhance student participation and active engagement, providing a more methodologically engaging and interactive approach to mathematics. The use of interactive tools and positive emotional induction in learning offers long-term benefits by actively engaging students and making learning enjoyable. These approaches improve memory retention, creativity and problem-solving skills by involving learners directly in the process (Scarlatos, 2006; Tang et al., 2022). Positive emotions reduce anxiety and increase motivation, creating a supportive environment that fosters confidence and sustained academic success (Lewis et al., 2009; Pekrun, 2014; Schweder et al., 2020). Over time, these factors contribute to deeper understanding, greater resilience and a lasting love of learning, laying the foundations for continued personal and academic growth. In particular, given the complex relationship between mathematical learning and emotions, it is even more important to design educational practices that prioritize students’ emotional well-being and optimize their learning experiences.

Finally, our findings can suggest practical steps for educators and policymakers to enhance learning outcomes. Educators can promote a positive emotional climate in the classroom by incorporating brief activities that boost students’ moods, especially in subjects like mathematics that often induce anxiety. Additionally, integrating interactive tools like SMARTER into the curriculum can increase student engagement and support diverse learning needs. Policymakers should consider investing in such technologies and providing resources and training for teachers to implement these tools effectively, promoting a more inclusive and engaging learning environment.

## Data Availability

The raw data supporting the conclusions of this article will be made available by the authors, upon request.
