# Supplementary material for: Emotions and interactive tangible tools for math achievement in primary schools
Source: Front Psychol. 2024 Oct 29;15:1440981. doi: 10.3389/fpsyg.2024.1440981 (PMC11554489; doi:10.3389/fpsyg.2024.1440981)
Supplement: Supplementary file 1 [file Data_Sheet_1.pdf]

Supplementary Materials

Figure S1

Photographs of the SMARTER device

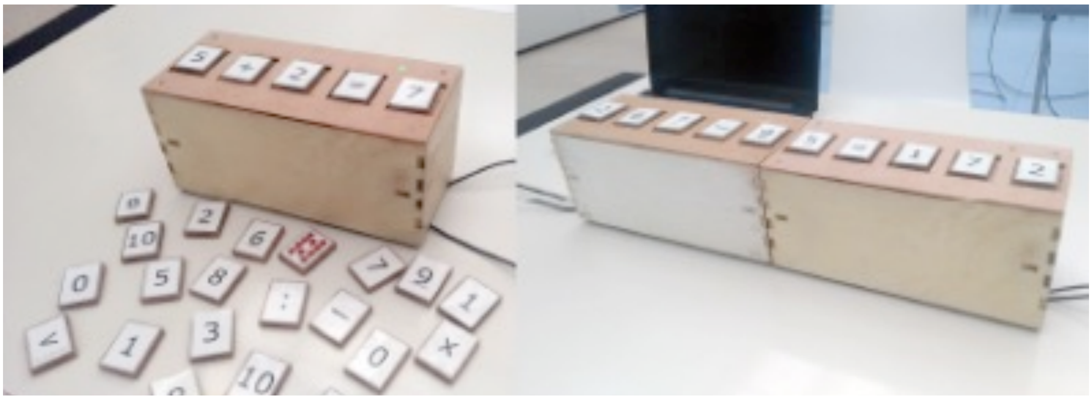

Note. On the left, a single device with plywood tiles representing numbers and mathematical operations; on the right, two SMARTER devices connected to realize a large working surface (from Andrao et al. 2022).

Figure S2

Schematic representation of the SMARTER components

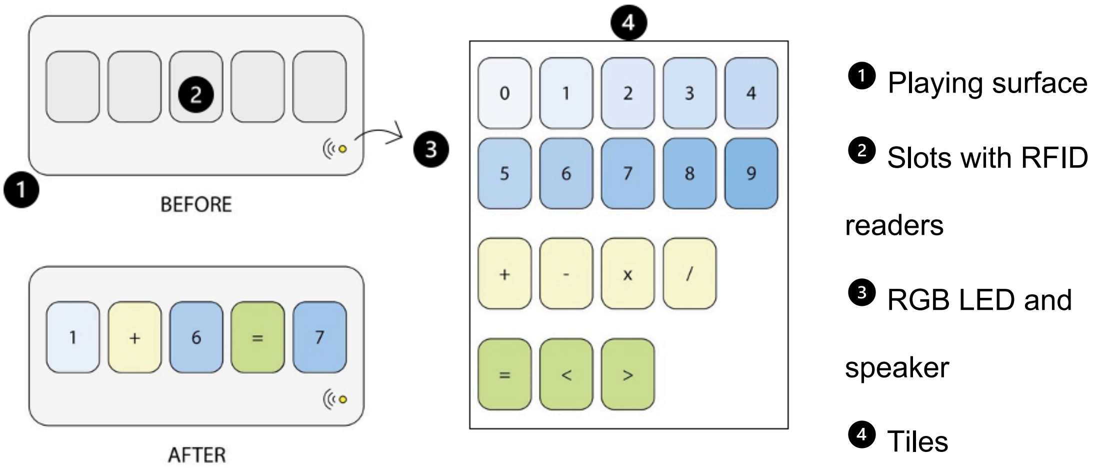

Figure S3

Photographs depicting the experimental setting

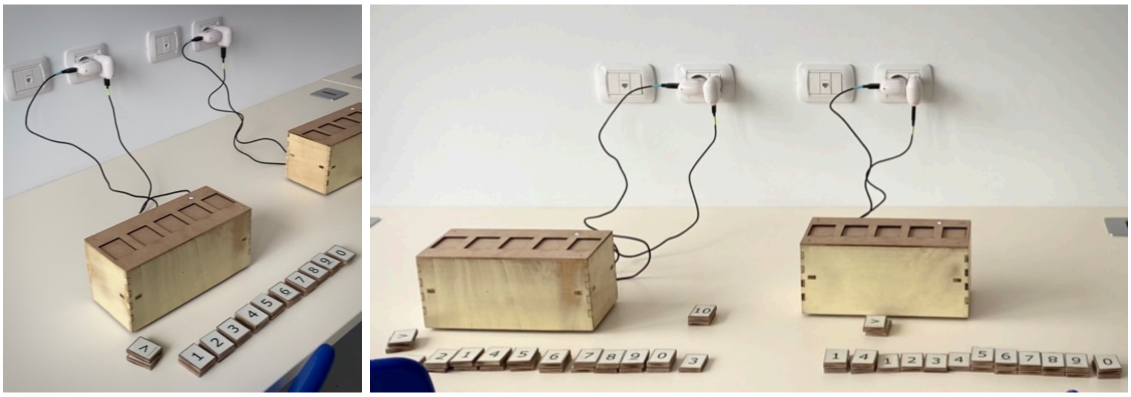

Table S1

Film clips shown for the elicitation of positive and neutral emotions during the testing phase

Valence	Video	Year	Extract	Start	End
Positive	*The Jungle Book* (Von Leupoldt et al., 2007)	1967	Song “The bare Necessities”	25:21	28:21
	101 Dalmatians (Essex et al., 2003)	1961	Birth of the puppies	15:20	18:20
Neutral	*The Last Unicorn* (de Freitas Brandão et al., 2016)	1982	Theme Song: unicorn and nature	0:00	3:00
	The Snowman (Talge et al., 2008)	1982	Theme song: children, winter and the snowman	0:00	3:00

Note. An additional film was shown to all children at the end of the experimental session in order to conclude the experiment with a positive emotional state. The film was Robin Hood (1971; start 50:55, end 53:55).
